# A paradigm-based explanation of trust

**DOI:** 10.1007/s11229-022-03993-4

**Published:** 2022-12-17

**Authors:** Friedemann Bieber, Juri Viehoff

**Affiliations:** 1 Department of Philosophy, University of Zurich, Zollikerstrasse 117, 8008 Zurich, Switzerland; 2 University of Manchester, MANCEPT, Oxford Road, Manchester, M13 9PL UK

**Keywords:** Trust, Paradigm-based explanation, Functionalism

## Abstract

This article offers a functionalist account of trust. It argues that a particular form of trust—Communicated Interpersonal Trust—is paradigmatic and lays out how trust as a social practice in this form helps to satisfy fundamental practical, deliberative, and relational human needs in mutually reinforcing ways. We then argue that derivative (non-paradigmatic) forms of trust connect to the paradigm by generating a positive dynamic between trustor and trustee that is geared towards the realization of these functions. We call this trust’s proleptic potential. Our functionalist approach does not only provide important insights into the practice of trust and its place in the broader web of social life, but also illuminates existing philosophical debates. First, pointing out how opposing theoretical accounts of trust each capitalise on only one of its functions, our paradigm-based approach reveals why they each contain a kernel of truth but are also deficient: the optimal realization of each function is tied to the existence of the other functions as well. Second, we show how a functionalist re-orientation can illuminate two recent disputes regarding (i) the question whether trust is explanatorily two- or three-place and (ii) whether (and to what extent) we can decide to trust others.

## Introduction

We sometimes face little difficulty in delineating social practices and defining the concepts that we use to refer to them. Think, for example, of the institution of marriage and the practices surrounding it. Yet when it comes to other social practices, especially those that are not defined and regulated by norm-setting institutions, delineating their boundaries and sharply defining our concepts can seem an intractable challenge. The difficulty of approaching a diffuse and multifaceted social practice with the standard tools of conceptual analysis is exemplified by the recent literature on trust: there are numerous, frequently conflicting accounts of what exactly needs to be true for* A* to trust *B*. Is the attitude that* A* needs to have a cognitive one (e.g. a belief), or is it a conative one (e.g. an intention to rely), an emotional one, or perhaps a combination of these? Does* A* need to do something to count as trusting over and above having this mental state? Can* A* trust *B simpliciter*, or only in certain regards? Not only are there competing accounts, but many of them seem plausible, explaining core aspects of our practice in genuine instances of trusting.

This article offers a novel, functionalist perspective on trust. We demonstrate that this approach can not only provide important insights into the practice of trust and its place in the broader web of social life but also illuminate existing philosophical disagreements. Pointing out how different theoretical accounts capitalize on one of the practical, relational, and epistemic functions of trust at the expense of others, our paradigm-based approach reveals why this is problematic: as the paradigm instance demonstrates, the optimal realization of each function is tied to, and to some extent depends on, the existence of these other functions as well. Another, more surprising point concerns disputed cases: we claim that conflicting intuitions about whether some episode of trust counts as ‘real trust’ are not a regrettable theoretical mess that needs tidying up in the form of definitional sharpening—to the contrary, ambiguity itself can be functionally valuable, for it allows us to ‘bootstrap’ different functional aspects of ‘paradigm trust’ when they are not yet warranted by facts. That trust has this proleptic character is itself part of what makes it valuable.

We begin our argument in Sect. 2 by briefly surveying two major rifts in the philosophical debate of trust and explaining why a paradigm-based explanation is particularly fitting for practices of this kind. In Sect. 3, we set out our paradigm form of trust—*Communicated Interpersonal Trust*—and introduce a specific scenario that instantiates it. In Sect. 4, we argue that our paradigm reveals three core functions of trust that serve, respectively, the practical, relational, and epistemic needs of beings like us and are mutually reinforcing. Moving to non-paradigmatic cases, Sect. 5 illuminates what we call trust’s *proleptic* and *anticipatory potential*: non-paradigmatic forms of trust can generate a positive dynamic between trustor and trustee that is geared towards the realization of all functions identified in the paradigmatic instantiation and can therefore qualify as forms of *trust proper.* In Sect. 6, we return to the two rifts in the literature and show how our account can explain them, explicating (i) how beliefs about trustworthiness, though important for justifying our overall practice of trust, are not indispensable for each successful episode of trust, and (ii) why, given the human needs that our practice answers to, there is no obvious explanatory priority as both sides of the disagreement about two-place vs. three-place trust claim. We thereby demonstrate how the paradigm-based account sheds light on existing disagreements. Section 7 concludes.

## Paradigm-based explanation and trust

Among the great variety of proposals in the philosophical debate on trust, two theoretical disputes seem especially deep. The first dispute concerns the question whether and to what extent we can voluntarily trust others. According to several influential proposals (Holton, 1994; McGeer, 2008; McGeer & Pettit, 2017), we can *choose* to trust others, and can do so even in light of (some) evidence that they are not trustworthy (either *tout court*, or in specific regards). That this idea is faithful to our linguistic practice comes out in such natural formulations as “I decided to trust her (to φ).” Yet other accounts maintain that beliefs about a trustee’s intentions and likely future behaviour constrain what can count as trusting others. “I do not believe that* B* will φ, but nonetheless trust her to φ” strikes some as a statement bordering on incoherence (Hawley, 2014; Hieronymi, 2008). While you cannot choose to trust against the evidence, they claim, you can *choose to pretend* to trust—acting *as if* you held the belief that* B* will φ—or *entrust* some φ-ing to* B* by rendering yourself vulnerable to *B*’s decisions regarding φ.^1^

The second dispute concerns the question whether trust has, at its core, a two-place or a three-place structure. According to several prominent accounts (Baier, 1986; Hawley, 2014; Hieronymi, 2008), explanations of trust should start from a three-place predicate *A trusts B to φ*, where “A” and “B” refer to agents and “φ” to some action or attitude. What we mean when we use the linguistically equally felicitous two-place predicate *A trusts B* is to be understood in terms of a non-articulated constituent: the semantic content of “A trusts B” is fixed by contextual aspects of the speaker’s situation. For example, when A is a client and B her lawyer, the unarticulated “φ” covers a range of actions set by justified expectations in the area of litigation but excludes more personal expectations like visiting her in the hospital when she is sick. Against this, recent accounts (Domenicucci & Holton, 2017; Faulkner, 2015) insist on a reversed explanatory relation: The most fundamental trust relation simply is the two-place relation *A trusts B* (a mere attitude) and three-place trust (an attitude that includes some act token) is explanatorily downstream—it is *because* we take the two-place attitude of trust towards a person that we trust her with regard to a set of specific actions.

We propose that the serious disagreement that exists on the nature of trust stems from the fact that the social practice which the concept tracks is essentially polyvalent, by which we mean that a number of important elements are jointly sufficient without any one of them being necessary for something to count as a genuine instance of trust. The considerable divergence of analytic definitions of trust stands as good testimony to this fact, we think, and speak in favour of at least complementing the standard analysis of trust. Moreover, even if one were to maintain that a set of conditions fully in line with our linguistic intuitions could be articulated, this would not on its own discredit the proposal of further pursuing our project of a functional explanation—for even if correct, these conditions may not be especially informative when it comes to the more demanding task of *explaining* the practice they pick out.^2^

We are not the first to cast doubt on the tenability of attempts to define trust in terms of necessary and sufficient conditions. Thomas Simpson (2012) for instance contends that “[t]he ways that the word [‘trust’] is used are simply too various to be regimented into one definition” (2012, p. 553) and proposes an alternative, genealogical study of trust (see also Faulkner, 2007; Jones, 2012; Kappel, 2014). Since our *paradigm-based explanation* resembles the genealogical method in certain respects, it is worth pointing out how our approach differs. Two differences, one methodological, one substantive, are crucial.

Methodologically, paradigm-based explanation aims to generate interesting conclusions without the kind of historicizing and/or fictionalizing to which genealogists resort (Fricker, 2021, p. 244; Queloz, 2020, p. 684). So, after laying out the paradigm form of the practice that we aim to investigate, we work out its *point* by dissecting the central purposes or functions that it serves those involved in the practice, and then see if other forms can plausibly be seen as derivative.^3^ One way to think about this is in terms of *difference-making*: What would be lost if we lacked the practice in our lives?^4^ Since the paradigm form of the social practice is one in which a great number of the social practice’s difference-making characteristics are present, we should be able to extract the central functions the social practice as a whole is *for*.

We conceive of genealogical analysts as both forerunners and allies in the project of explaining trust (its basic shape, internal norms of appropriateness, its outer limits etc.) in terms of the elementary functions that it serves us. But a paradigm-based explanation has the advantage of not relying on a narrative that can obscure our view. What we are interested in is, in the first place, not how we came to have a particular practice, but the ways in which it is relevant to us. Yet, as Fricker cautions, even in fictionalizing genealogies, the “narrative dimension of the fiction encourages one to mistake a deliberately fictional (or part-fictional) genealogy of X for a slap-dash attempt at a real history of X” (Fricker, 2021, p. 244).

There is a corresponding tendency of genealogical approaches to take rudimentary aspects of a practice like trust to be *uniquely* explanatorily central. This is evident, for example, in Simpson’s (2012, p. 557) attempt of deriving a form of “ur-trust” from a single human need that requires satisficing. It is a form of “rely[ing] on their freely cooperative behaviour” that, for Simpson, “constitutes the Ur-notion of trust” (2012, p. 558).^5^ Other, more complex forms—cognitive, conative, affective, and predictive trust—are then presented as derivative, catering to more complex needs.^6^ We are less convinced than Simpson that any single function of trust is more basic (in an explanatory sense) or prior (in a genealogical sense) than any other, so we rely on a central case in which those functions we claim exist are all realized.

This already points to the second, substantive difference between existing genealogical approaches and our paradigm-based approach, which is that we conceive of the paradigm case in terms of a successful *trust episode*. Existing genealogical stories proceed by identifying a core need on the side of the would-be trustor and then analyse how it is infused by additional meanings through contextual adaptation. But according to our paradigm case, there are important needs to be satisfied on both sides of the trustor/trustee relationship, and these come out clearest when the right sort of reasoning and the right sort of trust communication lead to practical, relational, and epistemic achievements on both ends. Our trusting practice has the shape it has as a result of both, our need to trust others *and* our need to be trusted.^7^

By taking as central the fuller episode of successful communicated interpersonal trust (CIT) that we believe is the contemporary paradigm form of trust, our paradigm-based approach allows us to provide a richer account of trust’s functions. Based on this, we explain *how* the non-paradigmatic instances are still instances of trust, whilst other practices in the vicinity (mere reliance etc.) are not. Put differently, our aim is not to provide a historical, or historicized, account of how we came to develop the practice of trust. Instead, we aim to show how a particular form of trust is paradigmatic—not because it came *first*, but because other forms of trust are explanatorily derivative: we cannot fully make sense of them absent the more central case of trust. In contrast to proponents of the genealogical approach such as Simpson, this leads us to emphasize a broader set of interlocked functions of trust: it not only helps us secure cooperation but allows us to build meaningful relationships and to improve our epistemic position.

## The basic form: communicated interpersonal trust

We start from the assumption that trust can take various forms and that no feature is necessary to all of them. Yet, we think that a particular form is *paradigmatic* because it reveals and rationalizes the characteristic functions of the practice of trust in a uniquely comprehensive and mutually reinforcing way and other instantiations are derivative in the sense of explanatorily relying on the basic form. As it obtains between two persons and is communicated, we label this form of trust *Communicated Interpersonal Trust* (CIT):

In the paradigmatic form, person *A* trusts person *B* if:

(i) *A* believes that in some domain D she can make legitimate demands on *B*;
(ii) *A* believes that, if communicated, her reliance on* B* in domain D will weigh with* B* as a substantial, non-instrumental reason for complying with her demands;
(iii) *A* relies on* B* and performs an act token from D; and
(iv) being *manifest*, *A*’s reliance communicates (i) and (ii) to *B*.

To render this proposal precise, a few terminological clarifications are in order. First, for our purposes, we understand a ‘domain’ as a mental file or ‘script’ that contains, in the simplest version, a dyadic set composed of trustor act tokens and matching trustee response tokens. As an example, consider the domain ‘work colleague’: It contains a set of trustor act tokens (“Attend faculty meeting”, “Submit student evaluation” etc.) and a matching set of trustee response tokens (“Show up on time for faculty meeting”, “Keep trustor’s assessment confidential” etc.). The idea of a domain reflects the fact that when we trust our colleague, some token pairs are salient whilst others—“Communicate birthday”, “Bring champagne” (Hawley, 2014, p. 3)—are not.^8^

Second, a demand is a moral claim on another person; insofar as it is *legitimate*, it gives rise to a directed *pro tanto* duty for that person.^9^ Third, we take it that to *rely* on someone to φ is to plan and act on the basis of the assumption that they will φ, and that to *manifestly rely* on someone is to rely on them in ways that render the reliance apparent to them (Hawley, 2019; Holton, 1994): a person can actively respond to being relied upon only where she realizes that she is relied upon. Fourth, for a fact to *weigh with* a person as a reason, we take it, it needs to enter their practical deliberation. Fifth, a reason is *substantial* if it warrants serious consideration and will often (though not necessarily) prove decisive.^10^ Sixth, a reason is *non-instrumental* for our purposes if it is grounded in the relationship between the trusting person and the trustee.^11^ If* A* believes that* B* would accede to her claim if she relied on her, but would do so exclusively for reasons unrelated to their relationship (say to impress *C*), then this does not satisfy the paradigm form. Finally, we take it that *A*’s manifest reliance can *communicate* that she holds the beliefs specified: her behaviour renders her act of reliance intelligible to the trustee as a communication whose representational content is the trustor’s belief in (i) and (ii).

In order to spell out the characteristics of the paradigm *form* of trust, and to later examine its function, consider a specific *case*.

*Secret*: In the course of a conversation about past relationships between two friends, Antonio and Betty, Antonio wonders whether to share a particular episode about an affair he had some years back. On the one hand, he cares to hear Betty’s views and judges it an entertaining anecdote. On the other hand, he has carefully guarded the story as he considers it embarrassing and would not appreciate it making rounds in his social circle. Based on Betty’s past behaviour, their companionship and her reputation, Antonio believes that if he were to manifestly rely on Betty to keep a story to herself, his act of reliance would constitute a substantial, non-instrumental reason for Betty not to share it. Moreover, he believes that, given their friendship, he can legitimately demand this. Antonio tells Betty his secret story, thereby communicating to her that he holds these beliefs.

*Secret* satisfies the conditions of our account of CIT. Moreover, we would naturally say that Antonio trusts Betty—or that he trusts her with a secret, or that he trusts her to keep secrets.

Having formulated and elaborated on a central form, and presented a specific case exhibiting it, we of course still owe a response to the question: Why believe that this form of trust is paradigmatic? In addressing this question, we first (Sect. 4) examine the functions of CIT to then (Sect. 5) argue that when any of its conditions is not satisfied, trust cannot serve these functions in the same comprehensive way, which we take to vindicate our proposal. At first sight, this argument may appear circular—first specifying a paradigm form of trust, then identifying the functions it can serve to finally argue that it is paradigmatic because it can serve these functions in a uniquely comprehensive way. We maintain that it is not. Taking a pre-theoretical understanding of trust as our starting point, we presuppose a basic grasp of what can reasonably be said to count as trust and what strikes us—despite all other disagreement—as common ground amongst theorists (e.g. that there is a difference between trust and reliance; that there is a difference between judging a person trustworthy and trusting her etc.). Then, in contrasting our proposal with others, which lack one or another of its conditions, we show that CIT qualifies as paradigmatic because it captures the functional richness of trust in a unique way. Moreover, it offers a method for ordering our intuitions about outlier cases, namely by identifying to what degree such non-paradigmatic instantiations still advance the central functions of trust. Given the disagreement about cases and intuitions, we think this is a legitimate strategy—and indeed a strategy fruitfully applied in relation to the analysis of other disputed normative concepts.^12^

## The point of communicated interpersonal trust

A social world in which we lacked the practice of trust would be one in which we would be missing important components of being effective rational agents, one which would make it difficult to form and uphold valuable personal relationships and attachments, and one that would deprive us of being optimally positioned to know and deliberate on the truth. It is for its ability to satisfy these three specific needs that we value and uphold (i.e. reproduce) the social practice of trust. This does not imply that each episode of communicated interpersonal trust will satisfy these needs—rather, a social practice of this particular kind serves us sufficiently well in central cases that we maintain it. Of course, in order to evaluate this claim, we need to (i) render more precise the particular needs we are picking out and (ii) explain *how exactly* the practice of trust enables us to satisfy these needs. This is the task we turn to now.

### Trust’s practical function: recruiting the agency of others

We live in a world where, in order to get what we want, we must effectively regulate our interactions with others: we must get them to understand what we aim for and motivate them to at least not act in ways detrimental to our plans. Of course, very often we need much more. As social beings with complex cognitive capacities, we can assess what motives others have and how they respond to adjustment of our actions and motives in light of what we take to be their interests and plans and vice versa (Jones, 2012, p. 63; Pettit, 1995, p. 212). One especially effective way of getting others to do what we need them to do in order to realize our ends is to communicate to them (verbally or otherwise) what we take to be the significance of our relying on them *for them*: “My plan to φ depends on your not failing to ψ, and given the importance I attach to φ, and taking into consideration the cost of ψ-ing for you, I believe that you ought to ψ!” How does this sketch of the dependency of our practical needs on the actions of others produce a positive answer to (ii), namely the question how the social practice of trust facilitates the satisfaction of said need? The general idea is that our social practice of trusting and being trust-responsive constitutes a powerful and effective instrument to manage our so being dependent.^13^

First, by actively placing trust in others (i.e. by relying on them in ways that communicate our belief that this should weigh with them as a reason), we can render it more likely that the other person will respond cooperatively to our dependency. ‘Placing’ trust in others here acts like extending a form of ‘credit’ to them. Through this ‘mechanism’, the social practice of trust can shape people’s intentions in ways that strengthen their willingness to act in cooperative ways that advance our practical aims. To illustrate this potential, consider the following continuation of our paradigm case that illustrates the ‘positive reinforcement dynamic’. Betty realizes that in telling her a secret, Antonio has trusted her and sees her as trustworthy to do as he relies on her. This motivates her to live up to Antonio’s trust. In turn, Betty’s positive response motivates Antonio to work her needs into his future plans in distinctive ways.^14^ We agree with Jones and McGeer and Pettit who elucidate this ‘empowering mechanism’.

In addition, we want to stress two further elements of our practice of trust that none of these authors discusses, but that are important to understand trust’s contribution to our ability to realize practical goals.^15^ Both elements draw on the implicit assumption that insights on the function of trust can be derived from comparing trust to normative powers and the reasons they furnish. Trust, we think, bears a resemblance to practical authority, and this is so *because* one of trust’s functions has a purpose comparable to that of practical authority, namely to serve the aims of the person over whom authority is held viz. the trustor.^16^

First, once we stand in a trusting relation to another person, that person’s actions and communications provide us with a particular type of reasons for action, namely second-order reasons, that shape our practical deliberation. And insofar as we are objectively justified in trusting the other person (i.e. insofar as we have placed our trust correctly), taking the actions and communications of the other person to generate reasons of this kind and acting on their basis does *in fact* enable us to realize our practical aims more effectively and efficiently.^17^ Let us say that in a variation of *Secret*, Antonio later learns that somehow the embarrassing episode has made rounds amongst his friends. That he trusts Betty provides him with reasons not to pursue certain avenues of inquiry, but to look for the leaker elsewhere. These trust-relation-grounded practical reasons are second-order reasons: they direct Antonio away from both considering and acting on certain considerations that would be warranted pursuing absent the trust-relationship. This point generalizes: if you trust, then absent strong evidence to the contrary, there is no need to spend time assessing the evidence that the trusted person breached your legitimate demands or failed to respond to your manifest reliance.

Second, to trust another person requires us to grant her discretion over what we care about.^18^ This, too, can be explained functionally in terms strikingly analogous to the case of authority: it is only *through* granting discretion to the agent in charge that we can reap the benefit of recruiting another’s ability, namely to track reasons that already apply to us. It is in this limited sense, we believe, that we should say that to trust somebody is to vest that person with a type of practical authority. And analogously to the standard explanation of when and why practical authority is justified (Raz’s ‘normal justification thesis’), we contend that trust, when justified, is in part justified because it facilitates our realizing our practical aims by granting discretion and acting on the second-order reasons that those whom we trust generate for us.^19^

### Trust’s relational function: attachments and meaningful relationships

One important difference between trusting another person and relying on her consists in the fact that the former, but not the latter, renders apt reactive attitudes: if the person (or object) on which we relied does not act as we anticipated, we may appropriately experience frustration, disappointment, or regret. Though these are also fitting where somebody we trusted has let us down, *more* is fitting in these cases, namely *reactive* attitudes: feeling wronged and betrayed, we blame others for their behaviour and deem these reactions appropriate. Given the connection between trust and legitimate demands we have postulated in the paradigm case, this should not come as a surprise—characteristically, blame is the appropriate response when we are wronged, and feeling betrayed is uniquely fitting when there has been an unanticipated breach of loyalty (O’Neil, 2012, 2017). Likewise, when we realize that we let down someone who had (justifiably) trusted us, we deem appropriate self-reactive attitudes like guilt and remorse. Despite the manifold disagreements, the fact that trust renders reactive attitudes apt in ways that mere reliance does not is shared by nearly all philosophers writing on the subject. Our suggestion is that we can make sense of this idea from a needs-satisfying perspective: the relevant attitudes that trust (but not reliance) renders appropriate are (quasi-)constitutively as well as contributively related to our human need to form attachments and navigate close relationships.

The first connection between trust and personal relationships is constitutive or quasi-constitutive: just as being somebody’s friend is closely linked to having certain emotions, desires, and dispositions to act towards that person,^20^ one cannot form close relations unless one adopts, at least in a broad range of conceivable situations, an attitude of trust towards that person. What explains this fact is the close connection between being a member in a valuable personal relationship and taking the participant stance towards others in that relationship, where the participant stance renders appropriate certain reactive attitudes and emotions.^21^,^22^

Although the difference between trust and reliance is typically framed in terms of the appropriateness of negative reactive attitudes like indignation and resentment in cases of breach, trust’s relational function can also be brought to the fore by reflecting on its effect on an ongoing relationship’s non-emotional features: A trustee’s breach of communicated trust provides the trustor with reasons to revise their relationship-constituting intentions and dispositions towards the trustee—whether or not they experience the emotions typically associated with betrayal.^23^

All intimate relationships presuppose trust; but clearly, not all trust presupposes an intimate relationship. In less intimate settings, trust plays a *contributory* role in facilitating loyalty and attachment. As Antonio and Betty repeatedly trust each other, they come to grow increasingly close. Perhaps what was initially just an acquaintance turns into an intimate friendship as each party further extends trust to the other. It is important that what they are responding to when this occurs is not just acting on the reason that another person is relying on them, but a reaction to another’s communication that a *particular* response to this reliance is called for. So, when things are transparent between trustor and trustee, doing as one is trusted to do can be characterized as uptake of the relationship-grounded claim implicit in the trustor’s communication.^24^ Suppose Betty repeatedly could personally gain by sharing the secret, but refrains. Moreover, suppose Betty does this *because it concerns Antonio, her friend*. It would then be appropriate for Antonio to feel gratitude. But Betty might also feel a certain gratitude towards Antonio. If she drew motivation from his trust, then he has helped Betty to prove herself as a trustworthy person. By contributing to our ability to transform, shape, and expand relational contexts not initially characterized by closeness, compassion, and intimacy into relational contexts where such norms are appropriate, our ability to trust answers to a fundamental human need of sociability.

### Trust’s epistemic function: knowledge formation and transmission

We now want to argue that, in addition, trust has an epistemic function that answers to our need to be effective deliberators and good knowers of the truth. Our suggestion is that being a successful epistemic agent consists in being endowed with certain capacities for knowledge formation and transmission, and trust plays a crucial role in this respect. Forming and transmitting true, justified beliefs is evidently important for instrumental reasons—we can cooperate and coordinate successfully with others only if we manage to deliberate with them and we can only orient ourselves and achieve our goals in the world if we manage to acquire knowledge or well-justified beliefs about it, much of it through receiving second-hand knowledge from others. But there is also intrinsic value to knowledge, and a related human need.

Being a good epistemic agent evidently requires good knowledge formation practices, such as paying attention to evidence and correctly adjusting one’s beliefs in light of it.^25^ But it does not end there: in a social world, epistemic agency demands capacities for knowledge transmission, both in the sense of being a competent transmitter of knowledge to others, and of being a competent recipient of transmitted knowledge from others (Greco, 2019, 2020). Our suggestion in this passage is then that through trusting and being trusted, we improve our epistemic agency, with regard to both knowledge formation and knowledge transmission.

On the first score: First, communicated trust involves a form of interaction with another person, and it leads, quite generally, to aligning our beliefs with others and to adopting better-justified beliefs—be they predictive beliefs about what people are like and are likely to do, normative beliefs about what we can legitimately demand of one another, or descriptive beliefs about what sort of relationship we have to other people. In our scenario, Antonio’s trust reveals to Betty that he holds certain favourable beliefs about her, that he believes that he can legitimately demand it of her to keep his secret to herself and that he takes them to stand in a not-too-distant-relationship. If Betty responds approvingly, communicating that she accepts Antonio’s demands and that she will respond by keeping the story to herself, they both acquire not merely more confident, but also better justified beliefs about what the other person believes and about what is likely to happen. In instigating such an exchange, well-placed trust can contribute to the alignment and improvement of our beliefs.

But even if Betty rejects Antonio’s trust, as long as she reacts openly and honestly, their interaction can serve a related purpose, namely that of improving joint deliberation. Suppose Betty tells Antonio that he cannot legitimately demand that she keeps the story to herself. Perhaps she considers it harmless, perhaps she considers it so severe that she feels obligated to inform his current partner. In either case, there is a conflict, which, if addressed, could be resolved in various ways.^26^ Antonio might come to accept that he cannot make a legitimate demand on Betty. Or his reasoning might convince Betty that he can make a legitimate demand after all. Or their discussion could give rise to a compromise, for instance that if Antonio informs his current partner, he can legitimately demand it of Betty to keep his story to herself. If they reach any such agreement, not as a result of threats, but by bringing about a convergence of beliefs, the episode of trust has helped them align their (normative) beliefs in a productive way. So, even where trust is disputed, it can improve their epistemic position.

Relatedly, our trusting practice, over time, improves our capacity to attune us to other people’s perspectives: if you trust a person to x, you make assumptions about the trustee, including their capacities, values and so on. Of course, we do not always consciously reflect on our trusting practices. But even where we do not, it is likely that our placing ‘trust bets’ in other people over time (sometimes more, sometimes less successfully) improves our general capacity to assess other people’s beliefs, intentions, and values. And conversely, when others communicate their trust in us (thus granting us a form of discretion over their affairs), we are thereby ‘charged’ with getting clear on what it is they have entrusted us with, which includes finding out about what they are like and what they want. Because our trusting practices create incentives to be trustworthy (and *trustor*worthy), parties are bound up in a system that utilizes our desire to seek acceptance and avoid negative emotional responses in order to improve our epistemic performamce. Attuning ourselves to others in these ways strengthens our ability to form justified beliefs.

*Knowledge transmission through testimony*: Much of the epistemic consequences of trust described so far are indirect. And perhaps our practice of trusting is not the only way of gaining advantages through attuning to others’ epistemic positions—but that doesn’t show that it is not significant.^27^ A more direct way in which trust advances our epistemic position relates to the second aspect of good epistemic agency, namely our capacity for knowledge transmission (and reception) through testimony, a field that has generated a sprawling philosophical literature.

There is a fundamental difference between, on the one hand, merely finding out that another person believes that p, and then using this information to support our belief that p, and, on the other hand, that other person *telling us that p* and us taking up and believing their assertion, thereby coming to form a justified belief that p. One important fault line in the literature on testimony is that between reductionist and non-reductionist accounts. According to the former position, testimonial knowledge can be subsumed under some other form, say, inductive knowledge. Anti-reductionists, by contrast, claim that other people’s telling us (their testimony) constitutes a unique source of knowledge.^28^ Anti-reductionist theorists of different variants offer diverging explanations of what grounds our permission to believe other people’s assertions. Amongst *social* anti-reductionist (as opposed to a priori reductionists [e.g. Burge]), several have argued for an intimate connection between trust and testimony by way of an assurance theory of testimony (Faulkner, 2011; Hinchman, 2005, 2017; McMyler, 2011; Moran, 2005). According to assurance accounts, “in telling A that p, S intends that A regards his intention that A believe that p as a reason for belief” (Faulkner, 2020, p. 331). Faced with S’s assertion, A’s coming to believe that p can be rational if (a) communications of this kind count as assurances, that is, as S’s assuming responsibility for A’s belief that p, and (b) S’s being trustworthy and A’s trusting S [with regard to p].

Thus, if we believe another person’s testimony, we trust her assertion that p to be true somehow directly, without pursuing all possible sources of evidence for or against p. Relying on other people’s testimony is clearly advantageous for us. Absent this practice, we would inevitably lack much of the knowledge we have about the world that does not result from direct experience. Trusting another person’s assertion displays yet again an authority-like structure of reasons: When a person we trust asserts that it is raining outside or that Timbuktu is not the capital of Mali, then this does not merely provide us with a reason to believe what is said, but also with second-order epistemic reasons to discount (some) evidence to the contrary: testimony provides warrant to believe. So, like in the case concerning practical decision-making, relations of speaker-trust (when we are justified in trusting the speaker) enable us to improve our epistemic situation by vesting a type of authority—in this case theoretical or expertise-authority—in another person. In this way, the practice of trust advances our epistemic and deliberative needs.^29^

We lack the space to fully articulate all the ways in which theorists have claimed that trust is essential for testimony—for example, John Greco has argued that transmitting knowledge inevitably requires trust by way of claiming that knowledge transmission is only possible where there is a form of *joint agency* characterized by cooperation and coordination between speaker and audience. Joint agency, in turn, is impossible absent trust; so trust is fundamental for knowledge transmission (Greco, 2019, p. 93). And drawing on Richard Moran, Katherine Dormandy has suggested that testimony not only requires trusting a speaker to the truth of p, but that it also requires trust on the speaker’s side that the audience recognizes and accepts them as a legitimate source of testimonial knowledge (Dormandy, 2020, p. 15). But we hope that there is sufficient evidence to prove trust’s importance in equipping us with the tools necessary for good epistemic agency.

More recently, trust’s crucial role for good epistemic agency *beyond* the issue of testimony has come into focus: Perhaps most explicit in this respect is the recent literature on epistemic blame and related practices (e.g. epistemic atonement) where several authors highlight the centrality of interpersonal trust for our epistemic agency in general (Boult, 2021a; Piovarchy, 2021; Woodard, forthcoming). According to one prominent explanation of the phenomenon of epistemic blame, we stand, as reasoners with the aim of forming true, justified beliefs, in a ‘general epistemic relationship’ to all others who share this aim (Boult, 2021b, 2021c). And not unlike other, more intimate relationships, our general epistemic relationship consists, amongst other things, of a trusting attitude that others exercise their epistemic agency well. Epistemic blame differs from the mere judgment that another epsistemic agent has failed to display good epistemic agency in that it consists in the withdrawal of epistemic trust from another person when that person’s epistemic behaviour falls short of the relational ideal. Importantly, trust’s relevance here extends beyond the issue of testimony, for we also epistemically blame others (that is, withdraw trust in their ability to act as epistemically trustworthy agents) when their epistemic practice exibits flaws with regard to non-testimonial features.

### Functional interactions in the paradigm case

Let us take stock: our practice of trust can enhance our agency by providing avenues for the pursuit of goals that would otherwise remain unattainable; trust enables us to form valuable relationships and navigate intimacy and personal attachments; and it facilitates knowledge formation and transmission by advancing our capacities for joint deliberation on practical matters, by aligning our beliefs and improving our epistemic position through testimony. Before moving on to non-paradigm instances of trust, we briefly highlight how the functions can be realized simultaneously, and in a mutually reinforcing way in the case of CIT. This is important because it is the fact that they are especially well realized that renders our account of CIT paradigmatic.

To approach this issue systematically, let us be clear about what we are claiming: Our paradigm case of trust is one where, recall, trust is interpersonal, entails beliefs about the propriety of what is expected and the trustee’s responsiveness, contains action in the form of reliance, and, finally, the act of reliance *communicates* the mentioned beliefs. Showing that our case is paradigmatic as far as these functions is concerned can logically proceed on two tracks, namely either, *positively*, by showing that these functions serve us especially well and complement each other in our central case, or, *negatively*, by indicating how scenarios in which any of these elements are missing fail to realize the described functions as comprehensively.^30^ The next section pursues the second track, but here we focus on the positive explication. Since we have stated three core functions corresponding to specific human needs, we want to indicate below at least three connections where, in the paradigm case, the realization of one function supports that of another.

First, consider the connection between trust’s ability to meet our practical and our relational needs in the communicated, paradigm case: In order to realize trust’s ability to enlist the agency of others, it is necessary to *rely* on them. Reliance, of course, is possible absent the beliefs necessary for trust and also absent communication of the relevant beliefs. But when you ‘manifestly rely’, i.e. when your reliance communicates the relevant beliefs, then you also engage trust’s relational function because the communicated normative expectations that advance the relationship are absent in the pure reliance case. Conversely, that is, viewed from a perspective of realizing our (relationship-independent) practical aims, we can say that between a social mechanism that ‘merely’ demonstrates to others how our aims depend on their behaviour and a social mechanism that does so by relating our second-personal stance to them (that, should they fail to come through, we will feel betrayed, are likely to revise our relationship-dependent attitude and intentions etc.), the latter will in many cases be more effective in recruiting their agency for our aims: normative expectations, when communicated, constitute a powerful, norm-governed sanction-mechanism because agents prefer not to be the object of anger, resentment etc.^31^

Second, consider the connection between the practical and the epistemic function of trust in the paradigm case. First, the practical ‘credit’ function of trust seems relevant too for epistemic performance. To the extent to which we have trust placed in us as valuable epistemic agents, we aspire to live up to this ‘credit’ (Faulkner, 2011, pp. 156–157). But because this mechanism works only if we realize that others hold such positive beliefs about our epistemic virtues, the explanatorily fundamental form here is one where the belief is communicated to the trusted agent. Moreover, we think that trust’s epistemic function is best served in the paradigm case, for it is in this case that the trustor does not merely hold and communicate a belief, but acts on his beliefs, thus relying on their truth. We would submit that it is a feature of successful joint deliberation that it not only facilitates agents’ exchanging their beliefs, but also communicating their strength—something that can be done well through action: one of the strongest things you can do to convey to your friend your belief that the glass in front of you contains water, not petrol, is to drink from it. Likewise, one way of credibly underlining the strength of one’s convictions (e.g. about one’s relationships) consists in relying on them whilst communicating this.

Finally, let us say a bit more about the connection between the relational and the epistemic function in the paradigm case: When we acquire knowledge through speaker trust in the form of testimony, we do in fact more than that, to wit, we are also, through believing what the other says (sometimes against the prevailing evidence), expressing our valuing the other as a sincere and trustworthy speaker. And this, obviously, can be both intimacy-forming and intimacy-sustaining. As the recent literature on testimonial injustice has shown, a failure to be recognized as a proper source of testimonial knowledge can undermine healthy interpersonal relations (Fricker, 2009). Moreover, and perhaps less obviously, *receiving* testimony too involves a relational dimension in that the speaker extends a kind of goodwill to the listener, namely one’s communicating that the other is a worthy recipient of testimonial knowledge.

The deep connection between trust’s relational and epistemic function also comes out if we take the idea of a ‘general epistemic relationship’ seriously. Like any relationship, our general epistemic one is governed by “a complex network of intentions and expectations that are oriented around our epistemic agency” (Boult, 2021c, p. 11364). The relationship is, in a sense, purposive: its mechanics and norms are aimed at equipping us to be good epistemic agents. But it is a *relationship*: when we realize that another’s epistemic practices have fallen short of the ideal’s norms, we revise our intentions and attitudes towards that person. When engaging in epistemic blaming, we do not merely judge that the agent we blame has fallen short of epistmic standards, but we are, in a sense, exercised by their failure. Even if these changes relate primarily to the other person’s shortcomings as an epistmic agent, it woud be hardly surprising if they did not also register in how we see our relationship to them with regards to more intimate matters. In other words, when you withdraw epistemic trust, you are also likely to alter personal attachments to that person.^32^

Based on this analysis of how the three functions of trust can reinforce each other, it is worth stating also what we do *not* claim.^33^ On the one hand, we do not claim that other practices could not also realize some of these functions. It is evident, e.g., that a wide range of practices—like asking one another for advice or circulating texts for anonymous review, but arguably also blaming or praising one another—can serve the epistemic function of aligning our beliefs with one another (including our beliefs about what the others believe). Similarly, emotionally taking part in another person’s life, being attentive to their desires and needs, or making thoughtful gifts, can all help us build closer relationships. And making an offer or request, or issuing a threat, are often successful means of recruiting the agency of others. Clearly, then, other practices can help us realize the three functions enumerated above—trust is unique only in being able to simultaneously realize them in a distinctly comprehensive and interlocking way.

On the other hand, we do not claim that non-paradigmatic episodes of trust could not also realize these functions, and sometimes all of them. The next section goes on to provide a careful analysis of the effects of each of the four conditions not being met. At this point, it suffices to address a potential objection. For it appears that even non-paradigmatic episodes of trust can realize the functions identified above and may sometimes arguably promise to do so more effectively. For instance, if I lack the belief that you are trustworthy, but then manifestly rely on you on the mere presumption that you are trustworthy, then—if all goes well—you respond favourably to me treating you as trustworthy. In this way, my behaviour may be effective at recruiting your agency and at jumpstarting the development of a closer, trusting relationship among us, revealing the “empowering potential” of trust (see e.g. McGeer & Pettit, 2017). Yet, while successful episodes of such *proleptic trust* are feasible, their success depends on, and is thus parasitic on, the existence of a broader practice in which the belief of trustworthiness exists. For if people only ever manifestly relied on others on the mere presumption of them being trustworthy, but never actually held the belief that they in fact are, then their manifest reliance would fail to have its desired effect: for it would then fail to signal a positive view of the other person, which in the proleptic case triggers the positive response. So, we do not claim that only episodes of CIT can jointly and comprehensively realize the three functions, but that this form of trust is unique in being able to sustain the realization of these functions.

## Proleptic or anticipatory: non-paradigmatic forms of trust

Moving on to non-paradigmatic forms of trust, this section discusses cases where (at least) one of the four conditions of Communicated Interpersonal Trust (CIT) is not satisfied. On the one hand, we argue that for each of the four conditions, if it is not satisfied, then the functions identified cannot be served as comprehensively under ordinary conditions. This, we argue, vindicates our claim that CIT is, in an important sense, paradigmatic. On the other hand, we show that even in cases where one of the conditions is not satisfied, the behaviour can have an important anticipatory potential—it may generate a positive dynamic that is geared towards the realization of the functions identified in the paradigmatic form. In some cases, namely where (i) or (ii) fail to hold, this potential is *proleptic*—in so acting, we can bring about what are preconditions of trust in its paradigm form. So, while other forms of trust are not paradigmatic, we suggest that we have reason to speak of *trust proper*; our aim, however, is not to demarcate boundaries of the term ‘trust’, but to employ our functional analysis to grade the centrality of purported instances of trust.^34^

In what follows, we address each of the four conditions in order, in each case examining an episode of trust where it fails to be met. Given the number of functions we have identified, the various ways in which individual conditions of CIT can fail to be met, and the multitude of ways in which episodes of trust can play out, our arguments in this section are bound to remain brief. While we cannot always discuss all potential objections in detail, we believe that our argument substantiates our main claims while conveying an overview of the manifold practices that, for functional reasons, plausibly fall under the heading of trust.

### Trust in the absence of a belief in legitimate demands

Recall the first condition of our account of CIT: *A* believes that in some domain D she can make legitimate demands on *B*. The condition fails to obtain if, to the contrary, *A believes that she cannot* make legitimate demands or if *A withholds belief* on whether she can make legitimate demands. We focus on the latter, weaker case. Consider an adaptation of *Secret*, where Antonio tells Betty his story, but withholds belief as to whether he can legitimately demand that she keeps it to herself. This could be for various reasons. Perhaps Antonio is uncertain whether Betty is a close enough friend and thus whether she is the sort of person of whom he could demand this. Perhaps Antonio has no doubts about his relationship to Betty but is morally uncertain whether one can impose such demands on friends. Presumably, in such cases, Antonio will believe that he can legitimately impose weaker demands on Betty—for instance, not to share the story for the fun of it or to inform him whether she intends to share it. So, one might think that if Antonio withholds belief on whether he can legitimately demand it of Betty to keep his story secret, he still trusts her, only in a more restricted domain. But this does not capture the idea that it makes a difference whether Antonio believes that he cannot make a demand on Betty at all or whether he merely withholds belief. In the latter case, but arguably not the former (cf. Sect. 6.2), Antonio’s acting as if he held the belief that he can make a legitimate demand counts as proleptic trust: in virtue of telling Betty his secret story and of the ensuing interaction between them, Antonio may alter their *relationship*, thereby making it the case that he now truthfully believes that he can make a legitimate demand on Betty and enabling Betty to recognize that Antonio makes a legitimate demand on her.

While this proleptic potential is significant, trust in its paradigmatic form requires *the belief* that one can make a legitimate demand. First, if Antonio merely presumes^35^ that he can make a legitimate demand, trust cannot serve the same epistemic function. If Antonio acts *as if* he believes that he can make a legitimate demand, then, even if this results in interactions that lead him to adopt this belief, he initially misleads Betty—so trust cannot here immediately align their beliefs. Second, if Antonio is uncertain whether he can make a legitimate demand on Betty, then, we think, he cannot confidently blame her if she fails to live up to the purported demand, because he could not claim that she did anything wrong—so reactive attitudes are not appropriate to the same extent. Third, the case of presumption is parasitic. Antonio’s presumption that he can make a demand on Betty will be effective at recruiting Betty’s agency in particular if she takes him to believe that he can make a demand, and she will interpret Antonio’s behaviour in this way only if people typically hold such beliefs. This is because if Betty does not take Antonio to believe that he can make a legitimate demand, she will not be motivated in the same way: for she must conclude that, in Antonio’s eyes, their relationship is not appropriately close or that his demand is excessive. But if we believe a demand to be misplaced, this generally diminishes our motivation to live up to it.^36^

### Trust in the absence of a belief that the other is trustworthy

Our second condition for CIT was this:* A* believes that, if communicated, her reliance on* B* to perform some act from S will weigh with* B* as a substantial, non-instrumental reason for complying with her demands. For shorthand, we may also say:* A* believes that* B* is trustworthy. This condition can be violated in two ways:* A* may believe that* B* is not trustworthy or withhold belief as to whether* B* is trustworthy. It is questionable whether we can speak of trust in the former case,^37^ but we again focus on the case of withholding belief.^38^ Suppose Antonio withholds belief as to whether Betty is trustworthy. If he nonetheless acts as if he held the belief, one might say that he merely *pretends to* trust Betty. Yet, we think that it is appropriate to say that Antonio trusts Betty because his behaviour can serve trust’s proleptic function. As before, if Antonio plans on and credibly acts in this way and Betty comes to think that he believes that she is trustworthy, then this may motivate her to keep the secret to herself and to reciprocate by telling Antonio secrets of her own, thereby setting off a sequence of interactions that lead Antonio to form the belief he initially lacked—that Betty is trustworthy.

Again, and for similar reasons, we think that this proleptic potential is important, but that in its paradigmatic form, trust requires a belief rather than merely the presumption of trustworthiness. First, if we merely presume, but do not believe that a person is trustworthy, trust cannot serve the same epistemic function. If Antonio acts as if he believes that Betty is trustworthy, then, even if this gives rise to an interaction that leads him to develop this belief, it cannot align their beliefs more immediately.^39^ Second, it is not obvious whether the same reactive attitudes are in order. One might think that Antonio cannot blame Betty to the same extent for failing to keep his secret; at least some of the blame seems to rest with him because, absent the belief that Betty is trustworthy, he took a risk.^40^ At the same time, one might think that Antonio can blame Betty even more—in presuming her to be trustworthy, he extended goodwill to her and she did not live up to it.^41^ Third, and most importantly, the case of presumption is parasitic on that of belief. Antonio’s presumption that Betty is trustworthy can generate a distinct motivating force, if she takes Antonio to see her as trustworthy, and she will interpret Antonio’s behaviour in this way only if people typically hold the corresponding belief.^42^ If people always acted as if others were trustworthy, but never actually believed them to be trustworthy, the practice of trust would collapse—it would lose its motivational force and people would no longer use it. So, while it is true that, in individual instances, acting as if we held the belief that someone is trustworthy may serve similar relational and practical functions as acting on the basis of an actual belief, this is so only because it is the norm that, in so acting, we hold the belief that the other person is trustworthy.

### Trust in the absence of manifest reliance

Recall the third condition of CIT:* A* manifestly relies on* B* and performs an action from S, where to rely on someone to φ was defined as planning and acting based on the assumption that they will φ. Evidently, if this condition is not satisfied, then the fourth condition cannot be satisfied either, for it presupposes that the trustor relies (and manifestly so). Relying on the trustee matters, because in relying we actually grant discretion and render ourselves vulnerable, and this is necessary for trust to effectively serve its practical purpose. Suppose Antonio believes that he can legitimately demand it of Betty to keep his story to herself and that she is trustworthy but does not tell her his story. This might be for various reasons, but simply assume that Betty has to run off before he gets a chance. Even if Antonio holds the specified beliefs about Betty, they remain rather toothless. It is by telling the story to Betty that he might get to hear her opinion, to signal his favourable views of her, and to build a closer relationship—but if he does not act on his beliefs, holding them will not produce these benefits. And even if Antonio were to communicate to Betty that he considers her worthy of trust, and would be happy to share secrets with her if given a chance, this will not generally have the same relationship-building effect. For it is by telling a carefully guarded secret, but not by simply asserting that he believes Betty to be trustworthy, that Antonio renders himself vulnerable. And it is by taking this risk that Antonio can credibly signal to Betty that he holds the belief that she is trustworthy.

Yet, even if this third condition is not satisfied, it is reasonable to speak of trust because the beliefs can, even in the absence of reliance, prompt a positive dynamic that encourages trust in the future. In contrast to above, it would not be apt to call this trust proleptic, as it does not help generate what are the preconditions of trust—the necessary beliefs are assumed to be already given and the reliance cannot be generated in a similar way. Still, it is *anticipatory* in the weaker sense of rendering trust behaviour more likely later on. Consider the case sketched above, where Antonio has formed the respective beliefs, but Betty leaves before he gets to share his secret, so he cannot rely on her to keep it to herself. Even so, trust can prompt a positive, anticipatory dynamic. First, the fact that Antonio has already formed the beliefs may render it more likely that he relies on Betty in the future—not only might he tell her the next time they see each other, but he might engineer such a meeting precisely because he believes Betty to be trustworthy. Second, he may communicate his beliefs in other ways, for instance by assuring Betty or their joint friends that he would always tell Betty his most guarded secrets. If Betty finds these assertions credible, they may in turn spark interactions that result in them growing closer together, thereby strengthening their willingness to rely on the other in a larger set of circumstances.

### Trust in the absence of a belief in successful communication

The fourth condition of CIT was that being manifest, *A*’s reliance communicates to* B* that* A* holds the beliefs specified in (i) and (ii). Suppose that while Antonio relies on Betty, his reliance does not communicate his beliefs that he can make a legitimate demand on her and that she is trustworthy. In one sense, this is not problematic. According to (ii), Antonio believes that his manifest reliance will weigh with Betty as a substantial and non-instrumental reason—and this does not require the communication of beliefs. Put differently, Antonio does not take Betty to respond to his trust, but to his manifest reliance. At the same time, whether Betty views Antonio as (merely) manifestly relying on her or whether she views him as trusting her makes a difference. This is so for three reasons. First, as we argued above, the view that another person is trustworthy will tend to have a motivational effect: it may encourage the trustee to live up to the high expectation and to reciprocate the trust, thereby generating a more powerful dynamic. Second, it is critical to establish agreement about the normative facet of the reliance. If Betty does not realize that Antonio, in manifestly relying on her, believes he can legitimately make a demand on her, she may not feel obligated to respond. Moreover, Betty may have an interest in not becoming subject to Antonio’s negative attitudes, which further motivates her to act cooperatively—but for this mechanism to be activated, Betty needs to see Antonio as trusting her. So, even if considering another person to be trustworthy is cashed out in terms of how one believes she will react to being manifestly relied upon, communicating to that person that one *trusts her* is valuable—generating greater immediate space for a positive response, it helps realize the functions of trust.

But the story does not end here. Ideally, there will be a common belief that Antonio holds the beliefs specified in (i) and (ii). For this to be the case, it is not merely required that Betty believes that Antonio holds these beliefs, but also that Antonio believes that Betty believes this and that Betty believes that Antonio believes that Betty believes this and so on (see Dougherty, 2019, p. 237). So, if things go well, Betty’s reaction also communicates to Antonio that she believes that he holds these beliefs. It is only by virtue of an ensuing back-and-forth of communication that Antonio and Betty can arrive at common rather than merely shared beliefs. This difference matters, for common beliefs put trustor and trustee in the best position to correctly interpret each other’s behaviour—as trusting rather than just relying, as responding to trust rather than to mere reliance, as taking the other person to respond to trust rather than to mere reliance and so on. (This also explains why, although in order to communicate his beliefs to Betty, Antonio neither needs to intend to do so nor take himself to do so, such self-reflection will typically be helpful as it allows Antonio to better evaluate Betty’s response.)

Of course, communication need not always be explicit. Whether communication takes place is to be evaluated against the applicable norms of interaction and the epistemic positions of the agents involved; often, silence may serve communicative purposes.^43^ If Antonio and Betty are close friends and it is evident that Antonio’s story is embarrassing, then telling the story to Betty might be sufficient for communicating that Antonio believes that he can make legitimate demands on Betty and that she is trustworthy. In fact, it might here be detrimental to assert these beliefs explicitly—if someone feels a need to make it explicit that she trusts us, where we take it to be evident, this may be alarming. It is for this reason that we require *A*’s *manifest reliance itself* to communicate that *A* holds these beliefs. If all goes well, we take it, no need for a separate, explicit act of communication arises.

Our claim that trust can unfold its motivational and relationship-building force to the fullest only where the trustee sees herself as trusted and that, for this reason, communication is important, ties in nicely with recent debates of normative powers such as promising, consenting, inviting, requesting, and commanding.^44^ In particular, the main argument why valid exercises of normative powers require communication also support our claim that, in the paradigm case, trust is communicated. To fully realize its functions, it is argued, consent requires common belief amongst the giver and receiver of consent, and common belief is hard to come by in the absence of communication (Dougherty, 2015, p. 237). Analogously, we claim that where it best serves its interpersonal functions, trust requires common beliefs and thus communication.

## An explanation for the theoretical disagreement on trust

We started our investigation by noting two deep rifts in the literature on trust. First, we noted the long-standing disagreement whether and, if so, to what extent we can choose to trust others. Second, we pointed to the ongoing dispute whether trust is at its core a two-place or a three-place relation. Having set out our account of trust, we close by returning to these theoretical disagreements. We believe that our functionalist account provides illuminating insights as to why these disagreements persist: different strands of the literature stress distinct functional elements we discerned in the practice of trust. Describing these positions in terms of the functions they emphasize permits a fruitful re-orientation of the debate. It provides an explanation of the roots of existing disputes and suggests ways in which they may be resolved.

### Cognitive vs. volitional accounts of trust

The first rift we noted in the literature concerns the question whether we can choose to trust. Some philosophers maintain that trust necessarily requires a trusting *belief* (Hieronymi, 2008; Keren, 2014), like the belief that the trustee will φ or the belief that the trustee is trustworthy. Following McMyler (2017), we may speak of *cognitivist accounts* of trust. Others insist that it is an important feature of trust that we can decide to trust (Faulkner, 2011; Hawley, 2014; Holton, 1994; Pettit, 1995), by which they mean we can choose to trust even when we lack the trusting belief. Following McMyler, we may here speak of *voluntarist accounts*. If, as is generally taken for granted, we cannot (directly^45^) choose what to believe, then cognitivist and voluntarist accounts are contradictory.^46^ As in Sect. 6.1, our aim here is not to settle the debate by defending one of these views; we instead aim to explain why both views contain a kernel of truth but remain unsatisfactory on their own, and to show how our paradigm-based account of trust offers a resolution of the existing dispute.

To start, note that one might think that in putting forward our account of CIT, we have sided with the cognitivists—for we have made it a condition that one does not merely presume, but holds a trusting belief. But while our account of CIT imposes demands on beliefs, it would be a mistake to conclude that we therefore align with the cognitivists. This would be a mistake because, in contrast to existing cognitivist proposals, we do not claim that *all* forms of proper trust require trusting beliefs. Instead, we allow for various non-paradigmatic forms of trust—some of which lack trusting beliefs, others their communication, yet others taking action on these beliefs—and we do not assert any clear hierarchy among them, let alone a categorical distinction. Insofar as they can all serve important functions of trust, they in principle all qualify as forms of trust proper.

In order for the distinction between cognitivist and non-cognitivist accounts to carry meaning and to generate a substantive dispute, one must insist that cognitivism requires a belief for all cases of true, or proper, trust. This is the view e.g. of Hieronymi (2008), who argues that we should draw a rigid line between *full-fledged trust* and *merely entrusting x* to another person. She reserves the notion of full-fledged trust for cases where (i) one believes that the other person is trustworthy (will do the thing you trust her to do) and (ii) one arrives at this belief in a particular way, namely in “something like the way that one might form a belief about one’s own future on the basis of one’s own practical reasons, given a background assumption about one’s own reasonableness and competency” (Hieronymi, 2008, p. 226). By contrast, one can entrust *x* to another person in the absence of such a belief, but to entrust *x* is not to properly trust.

Given our functionalist approach, we propose to grade the centrality of forms of trust by assessing their ability to serve certain functions. The functional perspective partly vindicates the cognitivists. It is, after all, for functional considerations that we require a trusting belief in the paradigm case. As we argued in Sect. 5.2, if one merely acted as if one held the belief that the other person is trustworthy, one’s behaviour could not serve the same epistemic function we value in trusting others; moreover, it is doubtful that the same reactive attitudes would be in order. In fact, while Hieronymi’s account is more demanding—we impose no restrictions on how the belief was formed^47^—she justifies the priority of full-fledged trust involving belief on the basis of similar considerations. Invoking the case of trusting another’s word, Hieronymi appeals to something akin to our epistemic function; and in noting that when you merely entrust something, the trustee may respond that you did not actually trust her, she invokes something close to our idea that different reactive attitudes may be in order, hence ‘impure’ trust does not develop the relational function to its fullest.^48^ So, the cognitivists capture an important idea that the voluntarists cannot account for: if we lack trusting beliefs, trust is deficient because it cannot serve all functions fully.

Where our account differs from Hieronymi’s is in allowing for proper trust in the absence of trusting beliefs. Our proposal to *not* declare a trusting belief a requirement for any form of *trust proper* is also motivated by functionalist considerations, but ones that in turn partly vindicate the voluntarists. In unpacking this claim, it is helpful to distinguish between two readings of voluntarism. On the naïve reading, the voluntarist notes that we can always choose whether to act on a given belief and insists that a mere belief is typically incapable of serving practical functions like that of recruiting the agency of others. While these two claims are correct, they fail to undermine the cognitivist view. The reason is simply that few cognitivists (for an exception, see e.g. Hardin (2002)) maintain that trust, in its purest form, requires nothing but a trusting belief. Being defined by the insistence that a trusting belief is necessary, and not that a trusting belief is sufficient, cognitivists are free to embrace additional requirements, such as acting on one’s trusting beliefs. On the informed reading, meanwhile, the voluntarist contends that we can trust others even in the absence of a trusting belief. This evidently yields greater discretion, as our trust behaviour is no longer constrained by our beliefs. It is this informed reading of voluntarism that stands in need of vindication.

But even in the absence of a trusting belief, important functions can be realized. As we have argued in Sect. 5.2, a presumption of trustworthiness can activate trust’s proleptic potential, enabling the formation of the trusting belief we initially lacked. Moreover, and independently, acting on the presumption of trustworthiness can help us recruit the agency of others. It is this latter, practical function that voluntarists like McGeer and Pettit (2017) rightly emphasize under the heading of “empowering potential”. There is an important sense, then, alluded to by voluntarists, in which important functions of trust can be realized even absent a trusting belief. Whereas Hieronymi remarks that “entrusting is what happens in the hard case, not […] in the best case” (2008, p. 219, fn. 10), once evaluated from our *functional* perspective, there is a sense in which Hieronymi’s “hard case” can be an especially good one—in managing to trust others absent the respective belief, we can leverage trust.

Our functionalist perspective suggests that there is some truth to cognitivism as well as to voluntarism—they both point to important functions of trust, none of which is obviously more important or central. At the same time, they go wrong in myopically attending only to their own insights. In its paradigm form, we have claimed, trust is cognitive—but beyond that, cognitive and voluntarist accounts of trust need not be seen as contradictory. There are non-paradigmatic forms of proper trust that involve trusting beliefs and others that do not, and they happily coexist. Yet again, or so we submit, our functionalist account offers not just a principled recipe for moving beyond the back and forth among linguistic intuitions (“Can you trust without relying?” “Can you trust me without believing I am trustworthy?”) but helps us to reconcile existing proposals in a more extensive framework that explains why we have a practice of trust in the first place.

### Two-place vs. three-place accounts of trust

The second dispute noted at the outset concerns the question whether trust is, at its core, a two-place or a three-place relation. At first sight, one might think that we side with the proponents of three-place accounts. After all, as we have characterized it, CIT contains three relata: the trustor, the trustee, and the domain in which the trustor trusts the trustee. But this first impression is mistaken. Instead, our domain-based account of trust falls in between. By incorporating the insight of proponents of two-place as well as proponents of three-place trust, it indicates a resolution of the existing conflict.

As we noted in Sect. 2, what is at stake in the dispute between two-place and three-place theories of trust is not which grammatical construct involving the verb “to trust” strikes us as central, but the *order of explanation*. As Faulkner puts it, “the fundamental explanation of why X trusts Y to φ is simply that X trusts Y or merely that X is trusting” (2015, p. 424).^49^ Domenicucci and Holton concur, suggesting that three-place trust is “the wrong place to start” and that “if we start with a three-place relation, and try to understand the two-place in terms of it, we will not succeed” (2017, p. 149). They draw on an analogy to intimate relationships to develop this point: statements like “I love her” are not well explained as implicit generalizations over “I love her sense of humour, I love her charm, I love her smile …”. Rather, “I love her” denotes an attitude that is irreducibly two-place (2017, p. 152). Likewise, they think, “*A* trusts *B*” cannot be explained as “*A* trusts *B* to [φ, λ, θ …]”. Other prominent authors (see e.g. Baier, 1986; Hawley, 2014) insist that, to the contrary, explanations of trust should always start from a three-place predicate that incorporates what one trusts the other to do. Where we use the two-place predicate, they insist, this third constituent remains unarticulated, but its semantic content is there, fixed by contextual aspects or the speaker’s situation. Yes, we might simply say that we trust the plumber or a stranger, but we have a highly qualified attitude of trust towards these people: we trust a plumber *to do their job*, we trust a stranger *to not run off with our laptop*. And the explanation of why a more general attitude was warranted frequently rests on rather determinate assumptions about what a person will do or what we can rely on her to do (“She was a doctor—I trusted her because I trusted her to give me the right medication!”).

We think that both sides are on to something and that our functionalist account is helpful in two ways: first, it offers a diagnosis of why we face the existing conflict and, second, it motivates a *domain*-specific account as a resolution. On the diagnostic point, we propose that some of the observations adduced to support different claims in this debate can fruitfully be expressed in functionalist terms—and that this helps us to appreciate their strength as well as their limitations. For reasons of space, we limit ourselves to two examples. First, consider the apparent impossibility of *fully* articulating the two-place attitude of trust in terms of a set of three-place tokens. We think that there is a functionalist explanation for this observation, which speaks against a general explanatory priority of three-place trust. As we argued in Sect. 3.1, trust serves its purpose of helping us to recruit the agency of others in part by granting them discretion over our affairs. But discretion cannot be formalized as a set of actions that are fully determined *ex ante*. If in trusting another, we were merely relying on them to perform some pre-set φ-s, then our practice would not leave room for the discretion that serves our aims.^50^

As a second example, consider the limits of the analogy between trust and friendship or love on which Domenicucci and Holton (2017) draw. That trust is sometimes genuinely two-place is well-explained by reference to the relational function it serves us. Trust best manages to establish intimate relationships amongst trustor and trustee when both understand it as the holistic appreciation—just as in the case of love and friendship, which we do not split into fine-grained spheres of interaction.^51^ But trust also differs from love and friendship. For while one cannot love somebody or be somebody’s friend for exclusively (or even predominately) instrumental reasons, we can trust others for purely instrumental reasons. The person who reasons “If we were friends, we could reap great [relationship-independent] benefits. So, let’s be friends!” is confused about the nature of friendship. But the argument “We could trade so profitably if there only was trust between us. Let’s trust each other!” is not based on a misunderstanding (though it does not capture all there is to trust either).^52^ Like love and friendship, trust answers to our need to form intimate relationships. But unlike them, trust also answers to our need to recruit the agency of others for (relationship-independent) purposes, for instance to achieve tasks that require cooperation. As a result, instrumental reasons can motivate trust, and the more a trust relationship is instrumentally grounded, the more salient are questions of competency and the ability to perform particular acts, which pushes us back towards a three-place conception of trust.

Our functionalist perspective also motivates our alternative reading, according to which trust is *domain-specific*.^53^ This is our positive—and more tentative—proposal. If trust’s discretionary element is incompatible with a narrowly conceived “*A* trusts *B* to φ”, *and* if trust’s practical role forces on us questions about the trustee’s competence and capabilities in such a way that explications of this attitude typically need to appeal to fairly determinate actions, then neither the three-place nor the two-place form seems explanatorily fundamental in all cases. The idea of a domain accounts for this—it resolves the conflict, allowing that whether the two-place or the three-place attitude of trust is explanatorily prior depends on the domain under consideration.

Our contention is that conceiving of trust as specific to domains leaves the conceptual space for the strategies proposed by proponents of two-place and three-place trust to each be adequate in *some* contexts. Despite its ostensible three-place structure, our domain-based account grows out of the realization that proponents of three-place and two-place trust are each onto something. When it comes to a repeated *Trust Game*,^54^ “Because I trust her!” is adequately explained in terms of a narrowly fixed set of expected reliance-responses and the two-place attitude seems fully reducible to *B*’s voluntary reciprocal behaviour in the past. But in a context of friendship, “Because I trust her!” is much better explicated in terms of a holistic attitude towards another person from which we might subtract some elements (e.g. “Not trust her to operate on me”) based on competency considerations. These are the limiting cases, most fall in between. Ordinarily, the area in which one trusts is restricted to an intermediate domain, where domains in respect of which we trust others can differ dramatically, in terms of breadth, but also in terms of *how holistic* an attitude of appraisal of the other person is presupposed. The domain-based account gives us the flexibility to account for this variation, while allowing for the explanatory primacy to fluctuate: sometimes, a more general attitude may best explain particular instances of trust, sometimes particular instances may best explain a more general attitude, and sometimes there may be no clear order of explanation.

## Conclusion

We started by noting two rifts in the existing literature on trust and then offered two observations. First, attempts to adequately capture trust in terms of necessary and sufficient conditions seem futile. Second, there are more and less central instances, as witnessed by core assumptions shared among theorists (that trust differs from reliance, that normative expectations and attitudes matter, that trusting a person is not the same as considering her trustworthy). In light of these observations, this article’s aim has been exploratory, namely to assess the potential of a non-standard methodological tool—a functionalist analysis in the form of a paradigm-based explanation—in examining trust.

Our analysis yielded three main functions of our practice in a paradigmatic instance, to wit, its ability to serve our practical needs to recruit the agency of others, our epistemic need to draw on the knowledge of others and engage in joint deliberation, and our relational need to establish and maintain valuable interpersonal relationships. We then investigated to what extent these functions are still advanced if some of the conditions defining the paradigm form of trust are not satisfied. We proposed that for functionalist reasons, we should opt for a liberal, ‘wide scope’ understanding of what counts as trust because many non-paradigmatic instances still have proleptic or anticipatory potential: they are still geared towards the realization of all the functions listed in the paradigm instance.

Our discussion is but a first sketch of how paradigm-based explanation might contribute to the burgeoning literature on trust. We are sure that even for those sympathetic to the project, there remains much to disagree with substantively: some will question our claim that the paradigm form of trust involves communication, others will challenge our argument that we should still speak of trust in some of the more peripheral cases. But if they raise such objections by drawing on an alternative interpretation of the practical, relational, and epistemic needs that trust helps to answer to (or indeed, by pointing to further functions that trust serves, which we have failed to identify), then we believe this project has already made a valuable contribution.

We also pointed out that thinking about trust in functionalist terms can shed new light on some of the disputes that have exercised theorists. Our focus was on two disagreements mostly internal to the nature and norms of trust and what should count as its explanatorily basic form. But there are exciting philosophical puzzles of trust beyond these. Important recent discussions deal, for example, with questions of when trust is warranted (trustworthiness) and the connection between trust and distrust.^55^ We are convinced that here too, functionalist approaches have valuable contributions to make—for example, the notable absence of three-place distrust is best explained, we believe, by the different purposes that trust and distrust serve social beings like us. But developing these ideas remains the task for another day.^56^

## Footnotes

1 The former is implied, for instance, by Katherine Hawley’s account of trust (2014, p. 10). The latter is how Pamela Hieronymi deals with cases of a voluntary nature (2008, p. 217).

2 Fricker (2016, p. 166). Joseph Raz (2001, p. 2): “to insist that conceptual explanations provide necessary and sufficient conditions is to concentrate excessively on the distinctive features of concepts, overlooking the importance of other features.”

3 Fricker (2016) pursues this approach in the context of blame, arguing that in the paradigmatic instance, blame needs to be communicated, thereby allowing for a realignment of the moral understanding of both parties. More recently, she argues that in the paradigmatic case, forgiveness requires remorse, its most basic point being to free the forgiver from a redundant feeling of blame (Fricker, 2021).

4 See in particular Queloz (2020, p. 684). Seana Shiffrin analyses our practices of promising and consent in a related *difference-making* way when she asks what would be missing in a world in which, though we had sovereignty over our body, we “had no ability to share or transfer these powers to others” (2008, pp. 501–502).

5 He notes “[in a world] where we raise families and enjoy living alongside each other, where others let us get on with what we are doing when it does not affect them, and where we can achieve things together that we could not do alone, I am routinely reliant on others’ free and cooperative action” (2012, p. 558).

6 A similar idea underpins Johnny Brennan’s (2021) proposal that *recognition trust* ought to be seen as the most basic form of trust, where recognition trust is “a latent expectation we have in others to acknowledge and respect our moral status as persons” (2021, p. 3800). For Brennan, recognition trust responds to a fundamental practical need that we have as agents who are dependent on others; it is only where recognition trust is given that other, more complex forms of trust can develop.

7 To contrast: Simpson’s ur-trust is fully realized where A willy-nilly trusts B and B betrays this trust. Our trust episode is closer to a successful interpersonal transaction. For a recent plea to analyse trust in terms of a successful performance, see Carter (2022a, 2022b).

8 Of course, trust also comes in varying degrees of strength, which are indicated by (i) the trusting person’s confidence in her belief about the trustee’s reaction to her manifest reliance, and (ii) how substantial a reason the trusting person believes her manifest reliance would be for the trustee.

9 The legitimacy is evaluated from the trustor’s perspective: a mafia boss who believes she can demand of an underling to take vengeance on her enemy could trust her in this regard, whereas the undercover agent who believes that she cannot legitimately demand this, could not. Moreover, we take it that the relevant beliefs can be held dispositionally.

10 Even if decisive, the reason does not require the trustee to do precisely as the trustor anticipates, but to discretionally respond to what one thinks the trustor would want one to do if she knew what one knows (see Sect. 4.1 on discretion).

11 We leave open the possibility that the relationship itself may be valuable primarily for instrumental reasons.

12 For instance, recent work on normative powers has involved exactly the move we are suggesting, namely that faced with clashing intuitions (sometimes about near-identical cases), disputes about conceptual centrality ought to be settled by appeal to which definition best realizes the core human purposes such powers are satisfying. Consider the question whether the mere mental state of consenting is sufficient for consent, or whether consent requires communication (Bolinger, 2019; Dougherty, 2015, 2019; Tadros, 2016). Those who argue that consent requires communication have stressed the resolution of moral ambiguity (Dougherty, 2015, p. 242) or the optimal management of moral risk (Bolinger, 2019, p. 188) whereas those defending mental-state views have stressed that their account best satisfies our interest in control (Tadros, 2016, p. 231). But all implicitly agree with our claim that whether or not something should count as φ-ing (or as a paradigm instance of φ-ing) turns on how well it serves some human need that consent meets.

13 As Jones puts it: trust helps us to “identify, in order to promote, a distinctive way in which human beings can actively and positively engage with the fact of another’s dependency” (2012, p. 66).

14 That trust can affect people’s intentions and conative attitudes towards others in these ways is, as McGeer and Pettit (2017) point out, ultimately a fact about human psychology: We are disposed to react positively to others holding us in high esteem. But neither does this diminish its practical relevance, nor does it in any way disvalue it—for the practice of trust we are concerned with is *our* practice, so it is only appropriate that it reflects our psychology. That trusting serves our practical aims as a ‘signalling device’ is compatible with the plausible observation that people who trust or respond to trust primarily with that mechanism in mind fall short of qualifying as ‘genuinely trusting/trustworthy’.

15 The reason is that Jones’ focus is on the concept of trustworthiness and the kind of responsiveness to trust that renders the trustee appropriately responsive. Our focus here is more generally about ‘trust episodes’ and their ability to satisfy needs of both trustor and trustee by generating ‘positive reinforcement dynamics’.

16 Here we rely on Raz’s model of authority which spells out the conditions of practical authority in terms of exclusionary (protected) reasons (1986, p. 36). We use the qualification ‘limited sense’ because, clearly, the phenomenon of practical authority entails aspects unrelated to trust relations, e.g. that authority creates *duties* for the person over whom it is exercised. Other authors have linked trust to a Razian account of practical reason (Keren, 2014, 2020; Simpson, 2017; Zagzebski, 2012). Keren in particular has explored in detail the analogous preemptive-reason-conferring aspects of (Razian) authority and trust (Keren, 2014, p. 2600).

17 For an account of second-order reasons, see Raz (1999, p. 39).

18 Domenicucci and Holton (2017, p. 151) equally stress this element.

19 Keren (2014, 2020) develops the thought that trusting another entails treating that person as providing us with preemptive reasons primarily with the aim of defending a doxastic account of trust (i.e. that trust entails a belief in the trustee’s trustworthiness or similar). Our functionalist discussion differs in that we propose to expound trust’s purpose in terms of the service that it provides to the trustor and in the emphasis on discretion.

20 As Samuel Scheffler pithily says, to value one’s friendship “just is, in part, to be disposed to treat that person's needs, interests, and desires as providing one with reasons for action, reasons that one would not have had in absence of the relationship” (2004, p. 247).

21 Of course, taking the participant stance towards another person’s behaviour is just a necessary condition for standing in a valuable interpersonal relationship: one might, after all, adopt a position of distrust that (if justified) renders appropriate negative reactive attitudes.

22 Explanations of how reactive attitudes constitute (near) indispensable elements in our ability to stand in valuable interpersonal relationships with others vary (Shabo, 2012; Strawson, 1993; Wallace, 1994). Moreover, there are different ways in which we may think about the second explanatory nexus to trust, i.e. whether it is the fact that the reactive attitudes can appropriately be held that *explains* that we are in the territory of trust or whether, conversely, it is the fact that we can trust that makes it appropriate to have these attitudes. Helm (2017) seems to defend the latter view, whereas the majority of philosophers writing on trust seem committed to the former. We need not take a stance here.

23 See e.g. Thomas Scanlon’s understanding of blame in terms of relationships-impairment (Scanlon, 2008, p. 131ff., 2013, p. 89).

24 Compare Holton’s climbing example: “I can have a reason for taking your hand that I do not have for taking the rope. In taking your hand, I trust you; in so doing our relationship moves a little further forward. This can itself be something I value” (1994, p. 69).

25 Some authors have stressed that developing these qualities is already tied up with developing forms of self-trust (Foley, 2001). We disregard this connection here.

26 Compare Fricker’s discussion of how communicated blame, even when rejected, can advance moral knowledge (Fricker, 2016, pp. 178–179).

27 Consider the case of blame: one central function of our practice of interpersonal blame, according to Fricker, is to bring about shared moral sensibility and understanding amongst blaming and blamed party (Fricker, 2016, p. 170). Thus, an instance of blame counts as more central to the extent that it serves this epistemic function. But surely blaming others is not the only way in which moral knowledge may be shared between victim and wrongdoer; the same can be achieved by victims informing wrongdoers of the harmful consequences of their actions without the characteristic ‘sting’ that marks out blaming, for example.

28 See Greco (2019, p. 95). Others have drawn the distinction differently (Faulkner, 2020; Simion & Kelp, 2020). What matters for our purposes is that (i) many see testimonial knowledge as grounded in considerations other than inductive inference and (ii) several authors see trust as playing a fundamental role of some kind in rationalizing our belief in other people’s testimony.

29 We recognize of course that the questions (i) whether testimonial belief amounts to trusting another’s assertion and (ii) how such trusting can amount to testimonial *knowledge* are enormously complex and heavily disputed amongst epistemologists. Since we cannot address these issues here, our reference to testimony is conditional: if, as the assurance theory of testimony suggests, testimony is formed through trust, and if this trust grounds knowledge, then testimony serves this part of the epistemic function we describe.

30 Although we do not focus on this here, it should be clear that trust that is not *interpersonal* realizes the relevant functions less well, and is, therefore, less paradigmatic.

31 Now, of course, the relevant mechanism here may also function if the trustor communicates normative expectations but merely *pretends* to hold them. But whilst this mechanism may sometimes realize both functions, the sanctioning elements would break down if the default assumption of communication—that the normative belief is genuinely held—would not hold (see our discussion at the end of this section).

32 The phenomenon of epistemic blame also reveals the centrality of communication: if the point of moral blame is to bring about shared moral understanding about wrongdoing, and this typically requires communication (Fricker, 2016, p. 173), then it seems plausible that epistemic blame too is paradigmatically communicated. We lack the space to develop this point further here.

33 We are grateful to an anonymous reviewer for pressing us on this point.

34 Others have emphasized that trusting a person can help render them trust-responsive and trustworthy, and have talked of the *empowering potential of trust* (McGeer, 2008; McGeer & Pettit, 2017). Building on these observations, Tsai (2017) has invoked the term “proleptic”, where he takes trust to be proleptic, roughly, if it is anticipatory—one does not really trust another person (yet), but acts as if one trusts her in the hope that this will render trust appropriate later on. We will understand the proleptic potential of trust in this sense: in certain contexts, trust can generate what appear to be its preconditions, namely the beliefs that one can make a legitimate demand and that the other person is trustworthy. See also Holton (1994, p. 63, n. 2).

35 We take it that to presume that another person is trustworthy is to take it to be (defeasibly) granted for the sake of practical deliberation that she is trustworthy. See Ullman-Margalit (1983).

36 This is not to say that it could not result in motivation: Betty might, for instance, be motivated to perform supererogatory acts or to behave in a way that signals to Antonio that she takes them, or wishes them, to stand in a closer relationship.

37 For instance, drawing on Holton (1994), Hawley has argued that I cannot rely on you—and therefore cannot trust you—to φ, if I believe that you will not φ (2014, p. 4).

38 Given our specification of (ii), *A* might withhold different beliefs here in different ways (e.g. whether the reason is judged to be non-instrumental, weighty etc.). Unfortunately, we lack the space to discuss the significance of different variations on how trustworthiness may be suspended, focusing on the general case.

39 An anonymous referee objects that a belief in Betty’s trustworthiness (CIT) does not improve trust’s epistemic function. After all, we can rely on other people’s knowledge even when not believing that they are trustworthy. So, worryingly, our paradigm case does not seem more central than this variation. But note that while one can believe what a speaker says without believing *the speaker,* believing the speaker by taking up S’s assurance that p (because you believe S to be trustworthy) and coming to believe p in part through this process of assurance provides an independent, powerful way for us to gain justified beliefs about the world. So, if, in an otherwise analogous case, we also held the belief that the person is trustworthy, then this would give us an additional, independent ground for inferring that p, namely S’s telling us that p. Therefore, the epistemic function is not as comprehensively served when the belief that the other person is trustworthy is absent/suspended.

40 Hieronymi suggests that breaches of trust are more blameworthy when you believe that the other person was trustworthy because “not only have you betrayed my confidence—i.e., told my secret—but you have also betrayed my confidence that you would not betray my confidence” (2008, p. 230).

41 A fact stressed by Domenicucci and Holton (2017, p. 154).

42 This is not to say that the presumption of trustworthiness could not be motivating in other circumstances. For instance, Betty might feel motivated to prove Antonio wrong, if she takes him to act on the mere presumption of trustworthiness while not actually believing her to be trustworthy. But in this case, the motivation has a different, and arguably less reliable source: for Betty might also decide to be content to simply confirm what she takes to be Antonio’s actual belief. By contrast, the belief that another person holds a positive view of oneself is more reliable in creating a strong, intrinsic motivating force to live up to it. We are grateful to an anonymous reviewer for alerting us to this further possibility.

43 Dougherty (2019, p. 246); see Langton (2007) for a discussion of the ambiguity of silence.

44 Enoch (2011) coined the term “robust reason-giving” to capture these and similar phenomena. We use “normative power”, but hope to be able to remain neutral between narrower and wider definitions (see e.g. Owens, 2012; Raz, 1972; Watson, 2009).

45 Arguably, we may decide to engage in behaviour which we know will over time shape our beliefs in certain ways. And of course, we can direct our thoughts at any point in time in such a way as to ‘make up our mind’ about whether to believe something (say whether B is trustworthy). But we cannot form a belief with a specific content by wanting to form that belief. Since a belief with a specific content (‘that B is trustworthy’) is what cognitivists take to be necessary for trust proper—its absence at most allows for the ‘as if’ mode of *entrusting*—we cannot decide to trust at will according to the cognitivist account (see Hieronymi, 2008, p. 217; Keren, 2020, p. 116).

46 But note that even if, *pace* the cognitivists, trust does not require beliefs, it may still not to be had for just any reason that shows it to be desirable.

47 Hieronymi relegates to the ‘impure’ side even cases where one both entrusts and has the belief in the trustee’s trustworthiness, if that belief is arrived at in other ways.

48 Many have remained suspicious of whether the distinction Hieronymi draws is as robust as she insists or as useful in distinguishing central cases of trust. Suppose, for instance, the trustee’s breach of your act of entrusting is very costly to you. Would the same response (“But you never really trusted me!”) not sound especially hollow in this case? The back and forth between these positions is captured nicely in Domenicucci and Holton (2017, p. 154). Also see the recent discussion in Carter (2022b).

49 In fact, Faulkner (2015) defends the further claim that one-place trust (“A is trusting”) is explanatorily more basic than two-place trust.

50 Domenicucci and Holton also stress the aspect of granting control (2017, p. 151), but do not draw the link to an authority-like mechanism we have described in Sect. 3.1.

51 As Jason D’Cruz observes, commenting on this analogy, we usually try to come up with ‘all-things-considered judgments’ about love/friendship, but not trust (2018, p. 248). Our added point is that this is so because of the practical function that trust serves us.

52 Our example is not meant to settle whether we can simply decide to trust at will or whether we can merely decide to cultivate trust. Rather, the point is that it is not inappropriate to decide to cultivate trust for self-interested, practical reasons. The same is not true, we would submit, for friendships and love relations.

53 We write “reading” because we hesitate to describe this as a genuine *third way* since we believe that the best interpretations of each of the prevailing positions could be read as implicitly endorsing a version of it. Two recent contributions that explicitly focus on domains are D’Cruz (2018) and Jones (2019). The first account that explicitly addresses trust as domain-specific is Jones (1996, p. 4).

54 See Brülhart and Usunier (2012). In a simple version of the Trust Game, person A can decide to transfer an amount of money to person B, where this amount is multiplied (by the bank), before B can reward A by transferring some amount of money back to A.

55 See e.g. D’Cruz (2019), Hawley (2014, 2019), and Jones (2012).

56 We would like to thank the audiences at the “Zurich-LMU Relational Normativity Workshop” at LMU Munich in 2019 and the colloquium at the chair in political philosophy at the University of Zurich. We would also like to thank Christian Budnik, Francis Cheneval, Matthieu Queloz, and Daniel Viehoff for feedback. Special thanks to the anonymous reviewers for this journal, whose constructive suggestions significantly improved the argument.
